# „Antibiotic-stewardship“(ABS)-Strategien in der urologischen Praxis und Klinik

**DOI:** 10.1007/s00120-020-01385-5

**Published:** 2020-11-17

**Authors:** U. Lichtinger, V. Greifenberg, A. Gessner

**Affiliations:** 1 Pädagogische Hochschule Vorarlberg, Feldkirch, Österreich; 2 Institut für Klinische Mikrobiologie und Hygiene, Universitätsklinikum Regensburg, Franz-Josef-Strauss Allee 11, 93053 Regensburg, Deutschland

**Keywords:** „Diagnostic stewardship“, Harnwegsinfektion, Antibiotikareduktion, Antibiotikaresistenz, Mikrobiom, Diagnostic stewardship, Urinary tract infection, Reduction of antibiotics, Antibiotic resistance, microbial, Microbiome

## Abstract

Antibiotika sind wirksame und sichere Arzneimittel und haben seit ihrer Einführung Millionen von Menschenleben gerettet. Die Weltgesundheitsorganisation (WHO) hat die weltweit zunehmenden Antibiotikaresistenzen als eines der größten Gesundheitsprobleme der Gegenwart identifiziert. Zu den häufigsten Indikationen für eine Antibiotikatherapie gehören Harnwegsinfektionen, bei denen nach aktuellen Erfassungen in einem sehr hohen Prozentsatz allerdings nicht leitliniengerecht behandelt wird. Um die fortlaufende Selektion von resistenten Bakterien, und unerwünschte oder sogar gefährliche Nebenwirkungen wie z. B. durch Schädigungen des Mikrobioms der Patienten zu vermeiden, sind dringend Strategien zur Verbesserung der Antibiotikatherapie durch „antibiotic stewardship“ (ABS) erforderlich. Insbesondere für Urologen in der ambulanten Patientenversorgung bedarf es hierfür neuer, innovativer und nachhaltiger Schulungskonzepte, die Wissen kontinuierlich aktuell halten und sachgerechte Antibiotikaverschreibungen nachhaltig unterstützen.

## Abstract (en)

Antibiotics are effective and safe drugs which have saved millions of lives since their inception. The World Health organization (WHO) has identified increasing antibiotic resistance worldwide as one of the greatest health problems of our time. The most common indications for antibiotic therapy include urinary tract infections, but according to current data, a very high percentage of these are not treated in accordance with guidelines. To prevent the continuous selection of resistant bacteria, and undesirable or even dangerous side effects such as by avoiding damage to the patient’s microbiome, strategies through antibiotic stewardship (ABS) are urgently needed. Especially for urologists in outpatient care, this requires new, innovative and sustainable training concepts that keep knowledge continuously up-to-date and support appropriate antibiotic prescriptions.

## Einsatz von Antibiotika

Im Verlauf der letzten Jahrzehnte ist weltweit ein stark zunehmender Verbrauch von Antibiotika zu verzeichnen [1]. Global nahm der Einsatz von Antibiotika pro Kopf der Bevölkerung von 2000 bis 2015 um fast 40 % zu. In den Industrienationen ist hierbei insbesondere ein Trend zur häufig nicht indizierten Verschreibung von breit wirksamen Reserveantibiotika besonders besorgniserregend. Der größte Anteil der Antibiotika wird nach wie vor in der Landwirtschaft eingesetzt. In der Humanmedizin werden in Deutschland Antibiotika zu >80 % von Ärzten im ambulanten Bereich verschrieben. Leider werden Antibiotika dabei nicht nur leitliniengerecht zur Behandlung schwerer bakterieller Infektionen, sondern auch prophylaktisch und in vielen Situationen empirisch bereits beim Verdacht auf eine Infektion eingesetzt.

Harnwegsinfektionen (HWI) gehören zu den häufigsten Gründen für einen Arztbesuch. Präzise Angaben für Deutschland zur Inzidenz fehlen aufgrund des oft selbstlimitierenden Verlaufs ohne Arztbesuch einerseits und der verschiedenen beteiligten Fachdisziplinen (Urologen, Hausärzte, Gynäkologen, Notfallpraxen) andererseits. Basierend auf nationalen und internationalen Studien ist davon auszugehen, dass mindestens jede 3. Frau eine oder mehrere HWI im Laufe ihres Lebens erleidet und jede 10. Frau sogar mindestens einmal jährlich betroffen ist.

**Figure 1:**
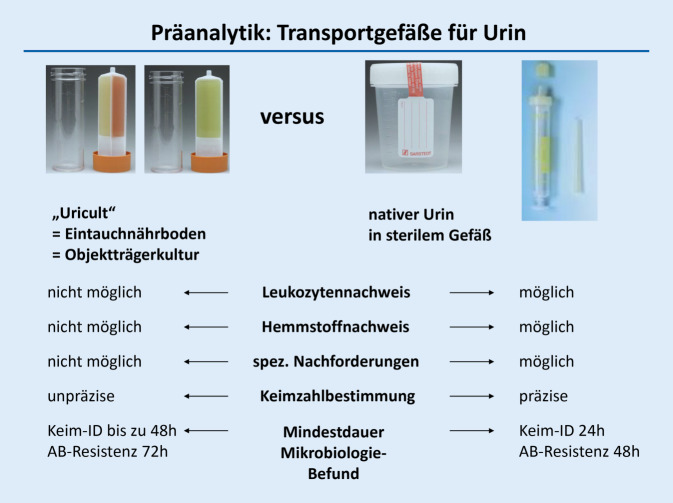

Insbesondere bei schweren oder chronischen Grunderkrankungen und rekurrierenden HWI-Episoden ist häufig eine Antibiotikatherapie indiziert und nicht vermeidbar. Anzustreben ist hierbei eine sachgerechte, möglichst vor(!) Therapiebeginn durchgeführte mikrobiologische Diagnostik, die besser als bisher die Präanalytik (Probenentnahme, Lagerung, Verschickung) im Sinne von „diagnostic stewardship“ (DGS) berücksichtigt. So sind sehr zeitnahe Untersuchungen von Nativurinproben sog. Eintauchobjektträgern eindeutig vorzuziehen (Abb. 1).

Bei rekurrierenden HWI-Episoden ist häufig eine Antibiotikatherapie indiziert

Die Antibiotikaverordnungen bei der Diagnose einer Zystitis (N30.0) auf Basis von Krankenkassendaten stehen dabei oft im starken Kontrast zu den Empfehlungen der Leitlinie von 2010 und der aktuellen Version von 2017 (AWMF-Register-Nr. 043/044). So wurden 2012 in 48 % der Fälle primär Fluorchinolone gefolgt von Fosfomycin und Cotrimoxazol (je 19 %) und Nitrofurantoin (10 %) verordnet. Sowohl Cotrimoxazol als auch Chinolone, letztere insbesondere zusätzlich aufgrund der Rote-Hand-Briefe vom April 2019 und Oktober 2020, sind jedoch nicht(!) als Mittel der ersten Wahl eingestuft. Offensichtlich werden also derzeit in Deutschland bis zu ca. zwei Dritteln der HWI nicht leitliniengerecht behandelt. Die Spontanheilungsraten der akuten unkomplizierten Zystitis liegen mit etwa 30–50 % nach einer Woche hoch. Die Therapie hat deshalb v. a. zum Ziel, die klinischen Symptome rascher zum Abklingen zu bringen und Komplikationen zu verhindern.

In einer aktuellen Studie wurde der Effekt einer primär symptomatischen Behandlung mit Ibuprofen mit einer sofortigen antibiotischen Behandlung verglichen. Unter symptomatischer Behandlung sind nach einer Woche 70 % der Patientinnen beschwerdefrei gegenüber 80 % bei antibiotischer Behandlung [2]. Ähnlich erfolgreich, was Symptomminderung bei HWI ohne Risiko von Sekundärkomplikationen betrifft, war eine größere Studie (CanUTI-7) aus dem Jahr 2018, in der die Effizienz und Sicherheit eines pflanzlichen Arzneimittels aus Tausendgüldenkraut, Liebstöckel und Rosmarin gegenüber einem Antibiotikum in einer multizentrischen, doppelblinden, kontrollierten und randomisierten Parallelgruppenstudie verglichen wurde [3]. Basierend hierauf kann Patientinnen mit einer akuten unkomplizierten Zystitis eine nicht-antibiotische Behandlung angeboten werden.

Für die Sicherheit einer HWI-Therapie ohne Antibiotika spricht auch eine aktuelle, im September 2020 veröffentlichte Studie aus England [4], in der über 280.000 HWI-Episoden bei fast 150.000 Patienten im Alter über 65 Jahren hinsichtlich des Risikos der Entwicklung einer Urosepsis analysiert wurden. Es zeigte sich kein erhöhtes Risiko für Komplikationen im Sinne einer Blutstrominfektion bei Verzicht auf eine Antibiotikatherapie.

## Problem 1: zunehmende Antibiotikaresistenzen

Resistenzen gegenüber Antibiotika bei Bakterien sind nicht neu – Gene, die Resistenzen gegenüber sehr neuen Antibiotika vermitteln, finden sich schon bei Jahrtausende alten Bakterien im Permafrost. Seit Einführung der Antibiotika sind bereits seit den 1940er-Jahren Resistenzen medizinisch relevant geworden, wobei sich klinisch relevante, resistente Bakterien jeweils fast regelhaft schon in den ersten Jahren nach Einführung eines neuen Antibiotikums beobachten lassen. Die Frequenz von Neuzulassungen von Antibiotika hat seit den 1970er-Jahren sehr deutlich abgenommen und die meisten Pharmahersteller haben sich inzwischen aus der Antibiotikaentwicklung zurückgezogen. Hierfür sind fast ausschließlich ökonomische Gründe verantwortlich. So verursacht die Entwicklung eines neuen Pharmakons durchschnittlich bis zur FDA-Zulassung (Food and Drug Administration) in den USA Kosten von 1,4 Mrd. US $ und nur ca. 14 % der Antibiotika in Phase-I-Studien erreichen die Zulassung [5]. Schätzungen gehen davon aus, dass ein Jahresumsatz von ca. 300 Mio. US $ für Hersteller die adäquate Schwelle für eine Profitabilität darstellt [5].

Da neu entwickelte Antibiotika jedoch möglichst lange als Reservesubstanzen und nur wenn unbedingt erforderlich eingesetzt werden sollen, sind diese Zielgrößen äußerst schwer erreichbar. Während in den ersten 50 Jahren des antibiotischen Zeitalters die Medizin ein für die Menschheit meist (noch) erfolgreiches Wettrennen – Resistenzentwicklung bei Bakterien gegen Weiterentwicklung von Antibiotika – erlebt hat, ist für die Zukunft eine sich öffnende Schere zwischen Bedarf für neue Antibiotika und entsprechenden Neuentwicklungen zu befürchten [5].

Schätzungen gehen davon aus, dass ca. 50 % aller Gaben von Antibiotika in der Medizin unnötig sind

Die Verbreitung von Resistenzen bei Bakterien erfolgt leider oft deutlich schneller als erwartet, wobei mobile genetische Elemente, die auch über Speziesgrenzen zwischen Bakterien ausgetauscht werden können, eine wichtige Rolle spielen.

Der wichtigste Treiber dieser Entwicklung ist der Selektionsdruck, der durch den Einsatz von Antibiotika ausgeübt wird. Schätzungen gehen davon aus, dass ca. 50 % aller Gaben von Antibiotika in der Medizin unnötig sind. Als Folge des steigenden Verbrauchs von Antibiotika in Humanmedizin und auch Tierzucht nehmen Resistenzen vieler humanpathogener Bakterien daher weltweit rasch zu und stellen nach Einschätzung der WHO eines der größten globalen Gesundheitsprobleme dar ([3], WHO-Bericht 2014). 2013 gaben die Centers for Disease Control and Prevention (CDC) in den USA bekannt, dass sich jedes Jahr mindestens 2 Mio. Amerikaner mit resistenten Bakterien infizieren, von denen ca. 23.000 an diesen Infektionen sterben. Auch in Deutschland sind multiresistente Erreger (MRE) v. a. in Krankenhäusern ein zunehmendes Problem: Bei schweren Infektionen mit resistenten Bakterien kann sich die Todesrate der Patienten im Vergleich zu Infektionen mit noch empfindlichen Bakterien durch die signifikant verzögerte adäquate Therapie verdoppeln. Die Lösung dieses Problems wird, absehbar wie beschrieben, nicht in der Entwicklung neuer Antibiotika liegen. Hier sind Neuzulassungen seit längerem rückläufig und auch das in den USA gesteckte Ziel, bis 2020 zehn neue Antibiotika zu entwickeln (10 × 20-Initiative) ist klar verfehlt worden.

## Problem 2: Mikrobiomschädigungen

Der menschliche Körper beherbergt auf allen äußeren und inneren Oberflächen komplexe mikrobielle Ökosysteme, auch in Bereichen, die früher als steril galten [6]. Viele der zahlreichen, sehr aktuellen Studien zum Mikrobiom haben hierbei den großen Einfluss der Darmbakterien, der sog. gastrointestinalen Mikrobiota, auf verschiedene Erkrankungen des Menschen gezeigt. Basierend hierauf sollten Auswirkungen von Antibiotikatherapien auf dieses komplexe Ökosystem zukünftig viel stärker berücksichtigt werden.

Mikrobiomveränderungen sind ursächlich beteiligt an der Entstehung unterschiedlicher Krankheitsbilder

Der menschliche Darm beherbergt eine enorme Masse (ca. 1,5 kg) und Anzahl verschiedenster Bakterien (über 3000 Spezies sind derzeit bekannt), Archaeen, Viren (Phagen), Hefen und Protozoen. Die Gesamtheit dieser Mikroorganismen und ihrer Gene wird als das „intestinale Mikrobiom“ eines Individuums bezeichnet. Mit bis zu 10^12^ Zellen/g Darminhalt im Dickdarm, dem am dichtesten besiedelten Bereich des menschlichen Körpers, tragen Bakterien den weitaus größten Anteil zu dem Genpool des mikrobiellen Ökosystems bei. Im Vergleich zum menschlichen Genom ist der Informationsgehalt des Mikrobioms mindestens um den Faktor 100 höher, weshalb inzwischen häufig der Begriff „zweites Genom“ („second genome“) gebraucht wird. Darüber hinaus zeichnet sich das Mikrobiom eines Menschen durch seine hohe Individualität aus. Während sich menschliche Genome nur in ca. 0,1 % ihrer Sequenz unterscheiden, wurden beim Vergleich humaner Mikrobiome >50 % Sequenzunterschiede zwischen Individuen nachgewiesen.

Das Mikrobiom etabliert sich nach der Geburt während der ersten Lebensjahre bis eine gewisse Stabilität und hohe Diversität erreicht ist. Zahlreiche Umwelteinflüsse wie die Ernährung, Erkrankungen, Infektionen, insbesondere aber Antibiotika können besonders in den ersten 3 Lebensjahren zu ausgeprägten, manchmal bleibenden Mikrobiomverschiebungen mit z. T. erheblichen Konsequenzen für die Gesundheit führen. So sind Mikrobiomveränderungen ursächlich beteiligt an der Entstehung unterschiedlicher Krankheitsbilder wie z. B. Adipositas, Diabetes, Allergien, chronisch-entzündliche Darmerkrankungen, verschiedenen Krebsarten, neurologischen und psychiatrischen Erkrankungen wie Depressionen und vielen anderen [6].

Die Veränderung des Mikrobioms unter Antibiose sowie dessen anschließende Erholung ist auch im Erwachsenenalter sehr stark abhängig vom verabreichten Antibiotikum und unterscheidet sich aufgrund individueller Mikrobiotaprofile von Mensch zu Mensch deutlich. Auch wenn die bakterielle Diversität des Intestinaltrakts meist nach mehreren Wochen wiederhergestellt ist, können Effekte auf einzelne Bakteriengruppen noch mehrere Monate oder sogar Jahre nach Absetzen der Antibiose nachweisbar sein [7]. Eigene Untersuchungen im Tiermodell zeigten z. B. starke Verschiebungen nach Gabe von Antibiotika, die zur Therapie von unkomplizierten Zystitiden vorzugsweise eingesetzt werden sollen (Fosfomycin, Einmalgabe oder 7 Tage Nitrofurantoin), die mehrere Wochen nach Absetzen der Therapie noch nachweisbar waren.

Unterschiedliche Effekte von Antibiotika auf die Mikrobiomzusammensetzung sind beschrieben, die gehäufte Infektionen (vaginale Mykosen,* Clostridium difficile*), Störungen der Immunbalance (Entwicklung von Asthma und anderen allergischen Erkrankungen) oder metabolische Erkrankungen (erhöhter Body Mass Index, Typ-2-Diabetes) begünstigen können [7]. Darüber hinaus bergen Antibiotikabehandlungen zusätzlich das Risiko der Anreicherung von resistenten Bakterien bzw. Resistenzgenen (Resistom) im menschlichen Mikrobiom [7].

## ABS zur Verbesserung der Therapie von Infektionen

Die Entwicklung neuer Antibiotika wird nicht ausreichen, um die wachsenden Resistenzprobleme beherrschen zu können. Ein optimierter Einsatz von Antibiotika ist deshalb ein zentraler und unverzichtbarer Teil der Strategie gegen die Resistenzentwicklung von Bakterien.

„Antibiotic-stewardship“(ABS)-Konzepte zum rationaleren Antibiotikaeinsatz werden seit 2 Jahrzehnten von Infektiologen, Mikrobiologen, Krankenhaushygienikern und Apothekern entwickelt. Die Ziele der ABS-Programme spiegeln sich alle im Ausdruck „steward“ wider, welcher sich vom altenglischen Wort „stigweard“ (Hallenverwalter) ableitet und so viel wie verantwortungsvolles Verwalten bedeutet. Aufgrund der Zunahme der Antibiotikaresistenzen wurden 2008 in Deutschland in der „Deutschen Antibiotika-Resistenzstrategie“ (DART) Ziele zur Bekämpfung der Antibiotikaresistenz in der Bundesrepublik festgehalten (Arbeitsgemeinschaft der Wissenschaftlichen Medizinischen Fachgesellschaften, 2013). Auch das 2001 erlassene Infektionsschutzgesetzes (IfSG) beinhaltete bereits diese Themen und verpflichtet seither zur Aufzeichnung von nosokomialen Infektionen und Erregern mit speziellen Resistenzen. Mit der Novelle 2011 wurde § 23 des IfSG um die Verpflichtung zur Aufzeichnung des Antibiotikaverbrauchs ergänzt. Basierend auf den aufgezeichneten Daten sollen Bewertungen und Schlussfolgerungen vorgenommen werden und daraus gegebenenfalls auch Konsequenzen gezogen werden. In der S3-Leitlinie „Strategien zur Sicherheit rationaler Antibiotika-Anwendung im Krankenhaus“ (Stand 31.01.2019) sind Empfehlungen zur Umsetzung dieser ABS-Programme und die ABS-Ziele beschrieben, die im Folgenden erläutert werden.

## Adäquate Antibiotikatherapie

Das erste ABS-Ziel ist die bestmögliche antimikrobielle Behandlung von Patienten mit Infektionserkrankungen und der angemessene prophylaktische Einsatz von Antibiotika. Zentral wichtig ist hierbei, die sog. „*4Ds*“ der Antibiotikatherapie zu beachten: „the right **d**rug**, d**ose, **d**uration and **d**e‑escalation“.

## Rationaler Antibiotikaeinsatz zur Minimierung der Resistenzentwicklung

Zweitens soll der übermäßige und nicht indikationsgerechte Gebrauch von Antibiotika verhindert werden, um die Selektion von Antibiotikaresistenzen sowie erhebliche unnötige Kosten für das Gesundheitssystem zu vermeiden. So werden z. B. immer wieder fälschlicherweise Patienten mit viralen Infektionen oder nicht-infektiösen Erkrankungen mit Fieber antibiotisch behandelt. Auch kommen bei ambulant erworbenen Infektionen viel zu häufig unnötige Breitspektrumantibiotika zum Einsatz, welche die Resistenzentwicklung begünstigen.

Um die Ziele des ABS zu erreichen, wurde durch die Gesellschaft für Infektiologie zusammen mit der Infektiologie Freiburg ein Fortbildungsprogramm für Ärzte und Apotheker ein mehrwöchiges, strukturiertes Fortbildungskonzept zum *ABS-Experten* erarbeitet.

Kernaufgaben von interdisziplinären ABS-Teams an Kliniken bilden die Erstellung lokaler Behandlungsleitlinien und Behandlungspfade unter Berücksichtigung lokaler Resistenz- und Erregerdaten und die Erarbeitung einer adäquaten Antiinfektivahausliste, welche kontinuierlich überprüft und ggf. angepasst werden sollen. Studien zeigten, dass durch diese Maßnahmen die Therapie- und Liegedauer sowie die Sterblichkeitsrate der Patienten reduziert werden. Sonderrezept- und Rezeptfreigaberegelungen, bei denen für die Verschreibung bestimmter Antibiotika (z. B. Breitspektrum- oder Reserveantibiotika) die Freigabe durch ABS-erfahrenes Personal erforderlich ist, ergänzen diese Maßnahmen sinnvoll.

Die zweite ABS-Kernstrategie umfasst informierende Interventionen. Dazu gehört die Gestaltung und Umsetzung von Fortbildungen, Schulungen und Informationen. Ziel ist es, im stationären und ambulanten Bereich Basiskenntnisse zur rationalen Antibiotikatherapie zu vermitteln und auf die ABS-Programme aufmerksam zu machen.

Ein optimierter Einsatz von Antibiotika ist Teil der Strategie

Die dritte ABS-Kernstrategie umfasst die Durchführung von Antiinfektivavisiten bzw. die proaktive Antiinfektivaverordnungsanalyse. Hierbei werden die Antibiotikaverordnungen im Hinblick auf (Verdachts‑)Diagnose, den mikrobiologischen Befund und den bisherigen Therapieerfolg vom ABS-Team mit dem Kliniker diskutiert und ggf. angepasst.

Die vierte Strategie ist die Integration des ABS-Programms in die Qualitätssicherungsstruktur der jeweiligen Klinik. Als geeignete Qualitätsindikatoren werden die Struktur (Infrastruktur des ABS-Teams), der Prozess (Antiinfektivaverordnungsverhalten) und das Ergebnis (Resistenz- und Antibiotikaverbrauchsentwicklung) regelmäßig bewertet und kommuniziert.

## Innovative ABS-Schulungskonzepte für niedergelassene Ärzte

Da alle aufgeführten ABS-Strategien und Maßnahmen für Kliniken konzipiert wurden, hat sich unsere vor einigen Jahren gegründete European Society for Antibiotic Stewardship (ESABS) zum Ziel gesetzt, ABS- und DGS-Konzepte für niedergelassene Ärzte zu entwickeln und anzubieten.

### Infobox 1 European Society for Antibiotic Stewardship (ESABS)

Die ESABS hat sich zum Ziel gesetzt, ABS- und DGS-Konzepte für niedergelassene Ärzte zu entwickeln und anzubietenhttps://esabs.net/
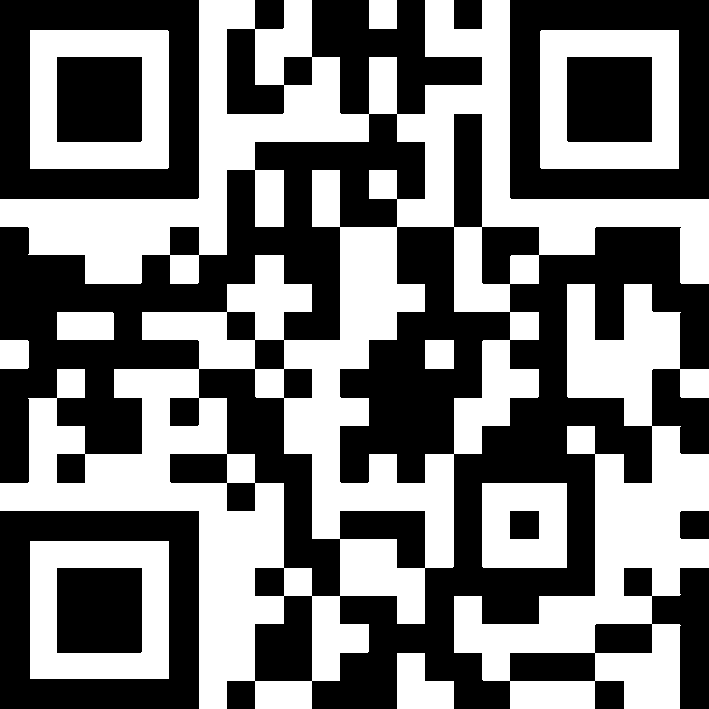


**Figure 2:**
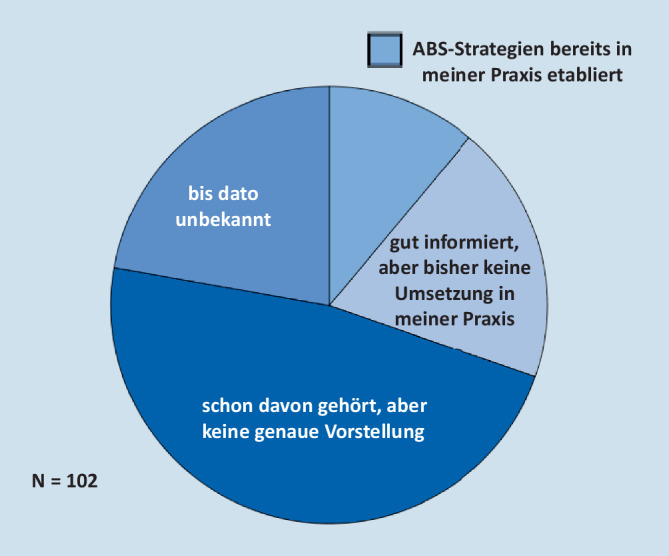

**Figure 3:**
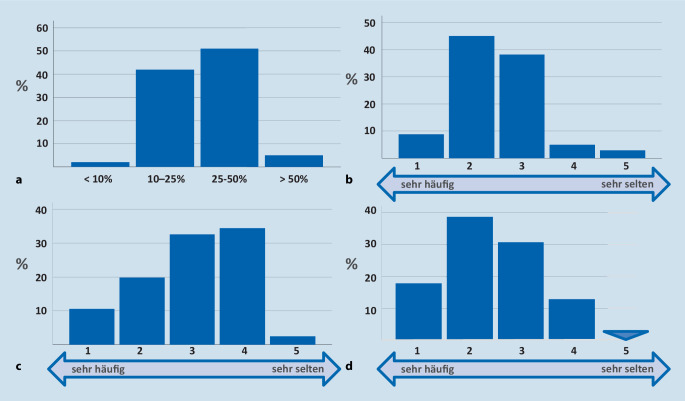

Unsere Befragung von über 320 niedergelassenen Ärzten verschiedener Fachrichtungen, darunter 102 Urologen, mittels standardisierter Fragebögen, ergab, dass grundsätzlich ein hohes Interesse an der sachgerechten Therapie von Infektionen besteht. Allerdings sind ABS-Strategien bei der Mehrheit der Urologen, vergleichbar zu Ärzten anderer Fachrichtungen, noch nicht bekannt oder nicht etabliert (Abb. 2). Ein hoher Anteil an Patienten stellt sich in urologischen Praxen mit Infektionsproblemen vor (Abb. 3a), von denen die meisten den Wunsch nach einer Antibiotikatherapie äußern (Abb. 3b). Korrelierend hierzu wird im Unterschied zu anderen Fachgruppen (Allgemeinärzte, Pädiater, HNO-Ärzte) eine relativ höhere Anwendungsdichte von Antibiotika angegeben (Abb. 3c, d).

Weder die dargestellten Leitlinien für ABS und DGS im Klinikbereich noch die für Kliniken entwickelten Schulungskonzepte können genügend hilfreiche Vorschläge für Hausärzte und Fachärzte in eigener Praxis liefern. Überwiegend im Alltag auf sich allein gestellt, brauchen diese verlässlichen Instrumente, die ein Konsil mit Fachkollegen ersetzen und in angemessener Zeit qualitativ hochwertig angewandt werden können. Diese umfassen:

- kompaktes, aktuelles Wissen zur Antibiotikaverschreibung,
- Verfahrensbeschreibungen mit klar definieren Aktionsabfolgen,
- konkrete Handlungsoptionen für die Praxis.

Ein Qualifizierungsangebot, das neben der Vermittlung aktueller Forschungserkenntnisse insbesondere auf eine Veränderung des Mindset im Kontext der Antibiotikaverschreibung abzielt, verfolgt zwei wesentliche Zielsetzungen: Information und Modifikation bisher bewährter Verhaltensmuster. Die Konzeption folgt dabei einer im Fortbildungsbereich trotz eindeutiger Forschungslage eher wenig verbreiteten Lehr-Lern-Auffassung und orientiert sich an einem gemäßigt konstruktivistischen Modell [8]. Das klassisch-traditionelle Lehr-Lern-Paradigma basiert darauf, dass Wissen eine Folge von Faktenlernen sei, von einer Person zur anderen direkt weitergegeben werde, und überlässt die Schließung der Lücke zwischen Theorie und Praxis dem Lernenden. Es bleibt stark in einer Präsentation von Wissen verhaftet. Im Blended-learning-Konzept von ESABS wird Lernen als konstruktiver, aktiver, selbstgesteuerter, sozialer und emotionaler Prozess verstanden [9], dem ein Angebot im Sinne von Denkmuster irritieren, Empiriewissen bereitstellen, neue Vorstellungen aufbauen, möglichst konkret anwenden und nachhaltig verankern folgt.

Es gilt, die Notwendigkeit zu einem „conceptual change“ bewusst zu machen und diesen anzubahnen

Lernende kommen mit ihren Erfahrungen und Annahmen, die zusammengenommen ihr Präkonzept zum Thema darstellen. Dieses ist wissenschaftlich betrachtet nicht immer oder vielleicht nicht mehr tragfähig. Es gilt, die Notwendigkeit zu einem „conceptual change“ bewusst zu machen und diesen anzubahnen [10]. Dabei ist es zunächst hilfreich, vorhandene Denk- und Handlungsgewohnheiten sichtbar zu machen und deren Chancen und Grenzen aufzuzeigen. So sollte zunächst bei den Teilnehmern gezielt nach bisherigen Behandlungsprozessen gefragt, die damit verbundenen Gedankengänge exploriert, die vorhandene Sinnhaftigkeit von Lösungswegen gewürdigt werden [10]. Die Dekonstruktion überholter oder falscher Wissensbestände erfolgt über empiriebasierte Informationseinheiten, die idealerweise von Experten aus der Wissenschaft dargeboten werden. Die bewusste Wahl der Akteure aus dem etwas anderen Professionskontext sorgt in der Regel für leichtere Akzeptanz. Ihre Distanz zum Feld darf allerdings gerade nur so groß sein, dass sie von den Fortbildungsteilnehmern als Personen außerhalb des eigenen Kollegenfeldes wahr- und gleichzeitig als Impulsgeber angenommen werden. Damit findet eine essentielle Handlungsperspektive Berücksichtigung: Multiperspektivität. Fortbildungsteilnehmer erhalten die Gelegenheit, verschiedene Perspektiven kennenzulernen und deren Verquickung als Bereicherung zu erfahren [9]. Unterstützt wird die kognitive Aktivierung durch ein Lernsetting, das „Lernende zum vertieften Nachdenken und zu einer elaborierten Auseinandersetzung mit dem Unterrichtsgegenstand anregt“ [11]. Weitere Lernphasen bieten kooperative Elemente zum Transfer des theoretischen Wissens in alltägliche Praxis. Das gemeinsame Durchlaufen eines Diagnose- und Verordnungsprozesses eröffnet einen ersten Raum, theoretisches Wissen mit Hilfe eines mehrstufigen Verfahrens in den konkreten Behandlungskontext zu übertragen. Durch Face-to-face-Interaktion und dialogische Lösung eines fiktiven Falles etablieren die Lernenden neue Kompetenz in sozialer Eingebundenheit [12]. Dieses Design in einer konsilähnlichen Situation bietet durch gemeinsames miteinander und voneinander Lernen Motivation – ein zentraler Einflussfaktor für hohe Lern- und Behaltungsleistung [13]. Nachhaltige Effekte sollen über Online-Tools unterstützt werden, die dahingehend wirken, dass die Praktiker mehrfach in Abständen an das neue Wissen und die neue Handlungspraxis erinnert werden.

Das ESABS-Fortbildungskonzept lässt sich zusammenfassend auf folgende Elemente verdichten:

- „conceptual change“ durch empiriebasierte Impulse,
- kooperatives Training konkreter Anwendungspraxis durch Beispielfälle,
- Etablierung des neuen Mindsets zur Antibiotikaverschreibung durch Online-Tools.

### Infobox 2 Weblinks

- S3-Leitlinie Unkomplizierte Harnweginfektionen:
  https://www.awmf.org/uploads/tx_szleitlinien/043-044l_S3_Harnwegsinfektionen_2017-05.pdf
- S3 Leitlinie Antibiotic Stewardship:
  https://fallreview.antibioticstewardship.de/dokumente/ABS_LL_S3_AWMF.pdf
- The Human Microbiome Project: http://hmpdacc.org/

## Fazit für die Praxis

- Antibiotika stellen ohne Zweifel eine der wichtigsten Errungenschaften der Medizin des 20. und 21. Jahrhunderts dar und haben viele früher potenziell tödlich Erkrankungen heilbar gemacht.
- „Antibiotic stewardship“ (ABS) ist essentiell, um die Wirksamkeit dieser wichtigen Medikamente zu erhalten, den zunehmenden Problemen mit resistenten Erregern entgegenzuwirken und Mikrobiomschädigungen zu vermeiden.
- Die Etablierung von innovativen und nachhaltigen ABS-Fortbildungen speziell für niedergelassene Urologen mit im Alltag praktikablen Konzepten für eine rationale Antibiotikatherapie hat hohe Priorität und ist eines der Ziele der European Society for Antibiotic Stewardship (ESABS).
